# Evolutionary algorithm-optimized feature fusion for accurate classification of shredded tobacco using multi-sensor data

**DOI:** 10.3389/fpls.2025.1728353

**Published:** 2026-01-12

**Authors:** Long Chen, Ni Tang, Xiao Wu, Yang Wang, Chuan He, Zongwei He, Lihua Xie, Xixiang Zhang, Xing Chen, Tao Zhou

**Affiliations:** 1 China Tobacco Sichuan Industrial Co., Ltd., Chengdu, China; 2 Biosensor National Special Laboratory Department of Biomedical Engineering, Zhejiang University, Hangzhou, China

**Keywords:** multi-sensor data fusion, feature-level fusion, genetic algorithm, shredded tobacco, GC-SAW, electronic nose, FTIR

## Abstract

**Introduction:**

Individual sensor systems have limitations in the complex task of classifying shredded tobacco. This study aims to overcome these limitations by developing a novel evolutionary algorithm-based feature fusion framework to enhance sensing accuracy.

**Methods:**

We fused data from three sensing modalities: GC-SAW, E-nose, and FTIR. A systematic comparison was conducted to determine the optimal fusion strategy. Seven dimensionality reduction methods were rigorously evaluated, leading to the selection of a genetic algorithm (GA) as the cornerstone for feature selection within our fusion framework.

**Results:**

Feature-level fusion was confirmed as the most effective strategy. The GA-based feature selection demonstrated exceptional performance, achieving a mean classification accuracy of 99.89% ± 0.79% across 50 independent test runs. This success stemmed from the algorithm's ability to intelligently distill the high-dimensional fused data into a compact, highly discriminative subset.

**Discussion/Conclusion:**

Our framework effectively balances information from the three sensing modalities to maximize their complementary strengths. This work confirms that evolutionary algorithm-based feature fusion is a powerful and robust method for unlocking the full potential of multi-sensor data, thereby significantly advancing the accuracy of complex plant material classification.

## 1 Introduction

Accurate and rapid discrimination of volatile organic compounds (VOCs) is essential in plant science, with important applications in monitoring food freshness (Shao et al., 2021), evaluating crop quality (Tatli et al., 2022), and tracking plant–pathogen interactions (Deng et al., 2022). The intrinsic complexity of real-world vapor mixtures, often composed of dozens of chemically similar analytes at varying concentrations and under fluctuating environmental conditions, presents a challenge for single sensing technology (Liu et al., 2025). Gas chromatography coupled with surface acoustic wave detection (GC-SAW) has long been revered for its exceptional separation capabilities and high sensitivity to specific functional groups, offering a powerful tool for identifying trace-level compounds (Chen et al., 2025). However, its utility in field-deployable, real-time applications is severely hampered by inherent drawbacks: lengthy analysis times due to chromatographic separation, the necessity for carrier gases and consumable columns, and vulnerability to column contamination or degradation from complex matrices (Meciarova et al., 2014). Conversely, electronic nose (e-nose) systems, which employ an array of selective chemical sensors combined with pattern recognition algorithms, have emerged as a promising alternative for rapid, *in-situ* analysis (Wang et al., 2022). These systems provide a holistic fingerprint of a sample’s headspace but are notoriously plagued by poor reproducibility (Liu et al., 2023a), drift over time (Chou et al., 2023), and acute sensitivity to ambient interferents such as humidity and temperature variations (Aurora, 2022). As a recent study demonstrated, even advanced e-nose arrays struggle to achieve discrimination accuracy when confronted with homologous compounds or under non-laboratory conditions, highlighting a critical performance ceiling for standalone systems (Liu et al., 2023b). Complementary to above vapor-phase analysis techniques, fourier transform infrared (FTIR) spectroscopy probes the fundamental vibrational modes of molecules, providing a definitive chemical fingerprint based on functional groups and molecular structure (Mallamace et al., 2018). Its reputation for high specificity and quantitative accuracy makes it a gold standard in both research and industrial laboratories for material identification and quality verification (Ferrari and Basko, 2013). For instance, in tobacco analysis, FTIR can rapidly authenticate origins and detect adulterants by characterizing bulk chemical compositions such as alkaloids, carbohydrates, and nitrate content (Fekhar et al., 2023; Marcilla and Berenguer, 2023). Nevertheless, the analytical strength of FTIR is also its primary operational weakness: it typically requires minimal sample preparation for solids and liquids but offers limited sensitivity to trace-level VOCs in the gas phase unless coupled with specialized accessories like gas cells, which increases system complexity and cost (D’Arco et al., 2022). Moreover, its performance can be compromised by overlapping absorption bands in complex mixtures and by strong signals from water vapor (Zou and Ma, 2014).

Thus, each analytical technology has strengths and weaknesses. GC-SAW delivers unparalleled separation and sensitivity for specific volatiles but lacks speed. The E-nose offers rapid, holistic screening but suffers from instability and limited specificity. FTIR provides robust molecular identification but is less suited for direct, sensitive gas-phase analysis of complex volatile mixtures. This technical challenge highlights respective limitations of single perception approach. Consequently, a paradigm shift towards multi-modal data fusion is not merely advantageous but essential to overcome the inherent constraints of single technology, synergistically combining their respective strengths to achieve a level of accuracy, robustness, and sensitivity that is unattainable by each alone.

Fusion methods are typically categorized into three levels based on the processing stage of integration, namely data-level, feature-level, and decision-level fusion (Ma et al., 2025). Data-level fusion involves the direct combination of raw or pre-processed sensor data streams before feature extraction is performed (Hassani et al., 2024). This approach aims to create a unified data set that preserves the maximum amount of original information. It has been successfully applied in areas like image processing (Zhou et al., 2023). However, its application to heterogeneous sensor data—such as combining chromatographic, cross-reactive, and spectral signals—is often problematic due to data heterogeneity, high dimensionality, and the presence of uncorrelated noise, which can collectively lead to the curse of dimensionality and obscure meaningful patterns (Castanedo, 2013; Romor et al., 2023). Decision-level fusion represents the abstract approach, where each sensing modality processes its data independently through a dedicated model to generate preliminary decisions or class probabilities. These individual outputs are then combined using rules such as weighted voting (Meng et al., 2021), bayesian inference (Griffiths et al., 2025), or dempster-shafer theory to reach a final consensus decision (Gao et al., 2021). This method is highly modular and robust to sensor failure, as evidenced in fault diagnosis systems (Zhang et al., 2022). By making decisions in isolation, this approach discards nuanced and cross-sensor correlations that may exist in the raw data (Mazher and Li, 2016). Feature-level fusion operates by first extracting important features from the raw data of each sensor independently. These feature vectors are then concatenated to form a composite feature set that is subsequently used for model training. This strategy strikes a balance between information preservation and data compactness. It has demonstrated significant success in applications like food quality assessment (Kiani et al., 2016) and disease diagnosis from multimodal medical data (Janakasudha and Jayashree, 2020), as it can capture complementary information from different data sources. The primary challenge, however, is the high-dimensional feature space. This requires effective dimensionality reduction or feature selection to prevent overfitting and ensure model generalizability.

Nevertheless, the promise of feature-level fusion is critically contingent on overcoming the challenge of high dimensionality. The concatenation of feature vectors from multiple sensors invariably results in a composite feature space where the number of features (*p*) vastly exceed the number of samples (*n*). This *p*>>*n* scenario not only risks model overfitting and impaired generalization but also amalgamates redundant features and sensor-specific noise, which can obscure the most discriminative signals (Silaich and Gupta, 2023). Therefore, effective dimensionality reduction (DR) or feature selection (FS) is not merely beneficial but essential for the success of any feature-level fusion framework.

While a multitude of DR algorithms—spanning filter (e.g., RF), wrapper (e.g., GA), embedded, and projection-based (e.g., PCA, LDA, PLS-DA, t-SNE) categories—are available, a definitive guideline for their application in fusing heterogeneous chemical sensor data is notably absent. Previous studies have often employed a single DR method in isolation, leaving a critical gap in our understanding: Which DR strategy is most capable of distilling a minimal, yet maximally discriminative and interpretable, feature subset from a high-dimensional amalgamation of chromatographic, cross-reactive array, and spectral data? The relative performance of these methods in this specific context, where features exhibit distinct information densities and noise profiles, remains unclear. This lack of a systematic, comparative framework hinders the development of robust and accurate multi-sensor systems for complex sample classification.

To address this methodological gap, our study moves beyond the conventional application of a single DR technique by presenting a rigorous, systematic comparison of seven representative DR algorithms for optimizing feature-level fusion of GC-SAW, E-nose, and FTIR data in shredded tobacco classification. The primary objective is to identify the most effective strategy for mitigating the curse of dimensionality in heterogeneous sensor fusion and to provide insights into algorithm selection. Our work makes several key contributions. First, we propose a three-tiered fusion framework that comprehensively evaluates and compares data-level, feature-level, and decision-level strategies. Second, we demonstrate, to our knowledge, the first concurrent fusion of data from these three orthogonal sensing modalities (GC-SAW, E-nose, FTIR) for this application, thereby constructing a more comprehensive digital fingerprint of the samples. Furthermore, we conduct a systematic investigation to resolve the high-dimensionality challenge intrinsic to feature-level fusion by evaluating seven distinct DR algorithms from diverse categories. Ultimately, we conclusively establish the superior efficacy of an evolutionary algorithm (Genetic Algorithm) for this task, demonstrating its unique ability to intelligently distill a compact, balanced, and highly discriminative feature subset. This approach effectively leverages complementary information from all sensors, achieving optimal classification performance while simultaneously enhancing model interpretability and robustness.

The remainder of this paper is organized as follows. Section 2 (Materials and Methods) details the tobacco samples, instrumentation for GC-SAW, E-nose, and FTIR, and elaborates on the data preprocessing steps, the three-tiered fusion framework, the seven DR/FS algorithms, and the classification models employed. Section 3 (Results and Discussion) presents the classification performance of individual sensors, compares the three fusion strategies, systematically analyzes the results of the seven DR algorithms, and provides an in-depth discussion on the optimal strategy. Finally, Section 4 (Conclusion) summarizes the main findings of this study, discusses the practical implications, acknowledges the limitations, and suggests directions for future research.

## 2 Materials and methods

### 2.1 Samples collection

**Figure 1:**
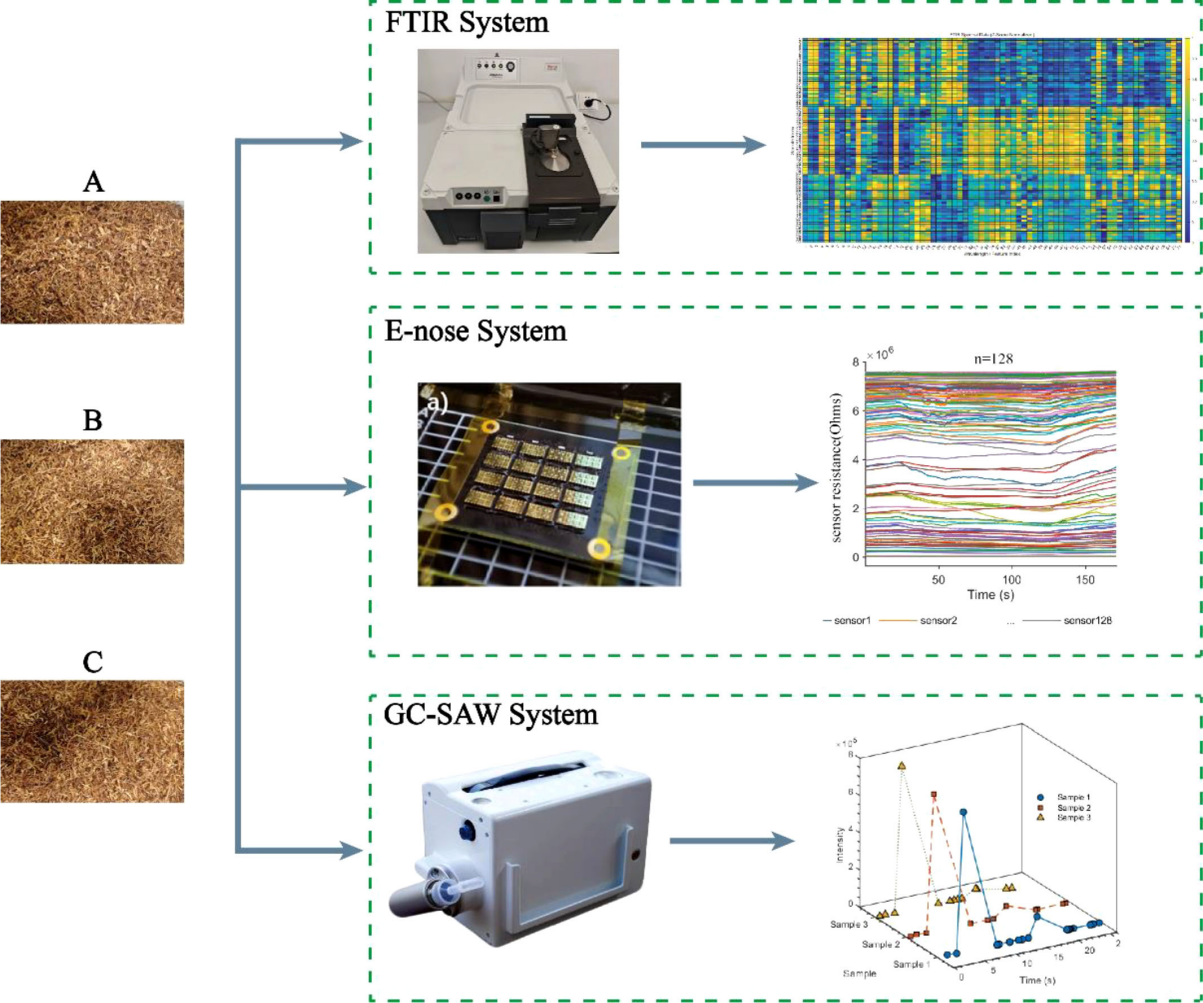
Experiment process of FITR, E-nose, and GC-SAW system.

The experiment samples were the shredded tobacco of the 3 different brands from tobacco factory in Chengdu city, China (**Figure 1**). A total of 90 samples were prepared for this experiment, with 30 samples from each of the three types of tobacco shreds. The three types of tobacco shreds were procured from three separate manufacturers and labeled them A, B, and C. All tobacco shred samples were analyzed using an electronic nose (E-nose), gas chromatography-surface acoustic wave (GC-SAW), and infrared (IR) spectroscopy. Samples were prepared according to the respective procedures for each technique: 0.5 g direct analysis for E-nose and GC-SAW, and 300 g powdered analysis for FTIR. This process generated raw data, which were subsequently preprocessed to form the final dataset comprising three matrices: GC-SAW (90 samples × 60 features), E-nose (90 samples × 128 features), and FTIR (90 samples × 71 features).

### 2.2 Electronic nose configuration and sensing principle

The electronic nose system utilized in this work was centered on a micro-electro-mechanical system (MEMS) gas sensor array. The array was composed of 128 distinct metal oxide sensors fabricated on a single silicon chip. Each sensor, functionalized with different sensitive materials (e.g., doped SnO_2_, WO_3_, ZnO), exhibits unique cross-sensitive characteristics to a wide range of VOCs. This design utilizes the principle of cross-selectivity, where the collective response pattern from the entire array is used for tobacco shred discrimination and classification, rather than relying on the absolute specificity of single sensor.

For the analysis, 0.5 g of each tobacco shred sample (S1–S90) was weighed and placed into a 50 mL headspace vial, which was then sealed and allowed to equilibrate for ~1 minute. The E-nose was connected to a computer operating data acquisition software. In the static measurement mode, the sensor array was first stabilized in clean air for ~60 seconds to establish a stable baseline. A sample vial was then introduced into the E-nose sensing chamber, and the baseline resistance was recorded as R_0_ (in Ohms). After ~120 seconds of measurement, the sample was removed, and the steady-state resistance was recorded as R_a_ (in Ohms). The sensor array was subsequently purged and rebalanced in clean air for about 60 seconds. The response intensity (I) of an individual sensor was defined as I = R_a_/R_0_, which is a dimensionless quantity (Ohms/Ohms) (Xu et al., 2025). Each sample was analyzed in triplicate, and the average of the three intensities was used for further data processing.

### 2.3 GC-SAW analysis

GC-SAW analysis is conducted in a strictly controlled, closed environment to ensure data integrity and instrument stability. The ambient temperature was maintained between 10°C and 40°C to prevent thermal drift in the SAW sensor’s resonant frequency and to safeguard the reproducibility of chromatographic retention times. Relative humidity was regulated at 20% to 70% RH (non-condensing) to avoid water vapor condensation on the piezoelectric sensor surface and electronic components, which could alter the baseline signal or promote static discharge. Furthermore, the atmospheric pressure was kept within 700 hPa to 1060 hPa to ensure consistent carrier gas flow rates, a critical factor for precise retention time accuracy. The entire system was housed in a clean, well-ventilated laboratory environment to minimize airborne contamination and prevent the accumulation of interfering vapors, thereby guaranteeing analytical reproducibility and operational safety throughout the experiment.

During the analysis, 0.5 g of each tobacco shred sample (S1–S90) was accurately weighed and placed into a 50 mL headspace vial, which was then sealed and allowed to equilibrate for 1 min. The GC-SAW system was connected to a computer running data acquisition software and was first stabilized in air for 60 s. In static measurement mode, the vial was introduced into the GC-SAW sensor chamber for analysis. After approximately 25 s of sampling, the vial was removed. Each sample was analyzed in triplicate, and the average of the three measurements was used for subsequent data processing. Based on the retention time of the target analyte across all samples, the start and end points of each chromatographic peak were determined. Each peak was then uniformly segmented into 60 intervals—a number chosen to balance the retention of sufficient peak shape information against the risk of feature dimensionality explosion. A schematic diagram of this process is provided in **Supplementary Figure 3**.

### 2.4 FTIR analysis

Near-infrared (NIR) spectroscopy was employed to characterize the samples based on their distinct light absorption properties at different frequencies. During measurement, each sample was exposed to a continuously frequency-scanned NIR beam, and the corresponding absorption signals were recorded to generate its NIR spectrum.

The instrumental procedure was as follows: the spectrometer was warmed up for over 30 minutes before use. The control software was then launched, and the spectral collection was configured with a wavenumber range of 10000–4000 cm^−1^ and a resolution of 8 cm^−1^. Approximately 300 g of tobacco shreds were ground into fine powder, and a representative portion was evenly transferred into a clean sampling cup. The powder was leveled and lightly compacted using a press under 20 g/cm² pressure, ensuring a sample thickness of at least 10 mm. The cup was finally placed on the motorized stage for NIR spectral acquisition. The concentrations of 71 key chemical compounds were directly obtained from the processed NIR spectral data using the instrument’s proprietary quantitative software (tobacco near infrared big data system platform). All samples were processed through an identical and automated pipeline within this software to ensure consistency.

### 2.5 Framework of data fusion strategy

#### 2.5.1 Data-level fusion

**Figure 2:**
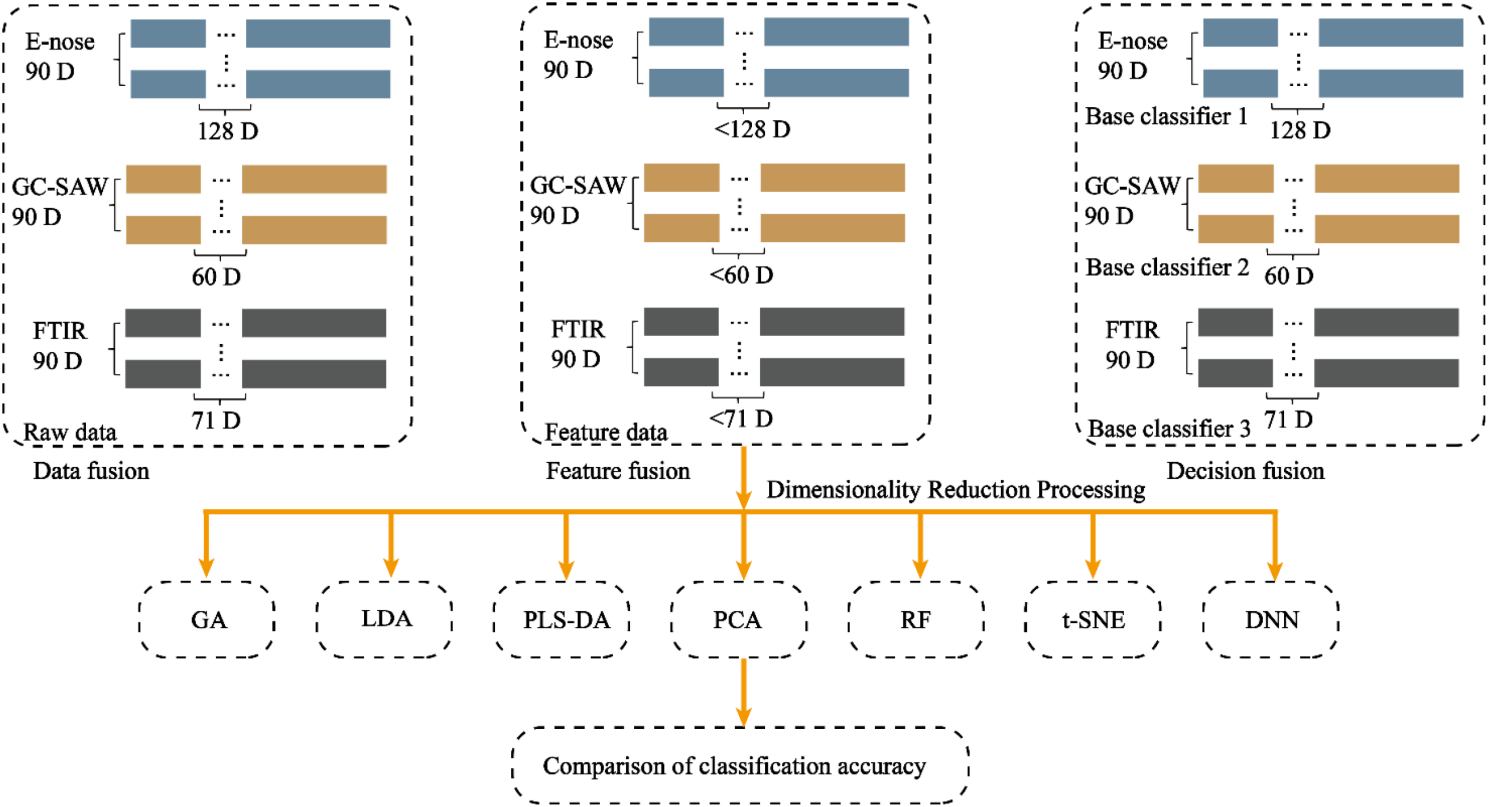
Schematic diagram of fusion methods toward the data of FITR, E-nose, and GC-SAW system.

Data-level fusion, also referred to as low-level fusion, was implemented by directly concatenating the raw feature vectors from several modalities (Lahat et al., 2015). In this study, the respective data matrices from GC-SAW (60 features), E-nose (128 features), and FTIR (71 features) were combined for each of the 90 samples, resulting in a single, comprehensive data matrix of dimensions 90 × 259(**Figure 2**). This fused matrix, which integrated the raw, low-level information from all sensors, was then used to train and evaluate the classification model.

#### 2.5.2 Decision-level fusion

A decision-level fusion strategy was employed to effectively integrate information from the multiple data sources. Individual support vector machine base classifiers were first trained separately on the standardized feature sets derived from GC-SAW, electronic nose, and FTIR spectroscopy. Within a leave-one-out cross-validation framework, each sample was sequentially used as the test set while the remaining samples trained the models. For each test sample, the three base classifiers provided not only a class prediction but also the associated probability scores. A weighted voting scheme was subsequently applied for decision fusion, wherein the probability scores from all three classifiers were summed, and the class with the highest aggregated score was assigned as the final fused prediction.

#### 2.5.3 Feature-level fusion

A feature-level fusion strategy was adopted to construct a unified feature space from the multi-sensor data. To eliminate redundant information and enhance feature discriminability, seven distinct dimensionality reduction techniques were systematically evaluated and compared. These methods encompassed: 1) filter-based approaches, including random forest (RF) for feature importance ranking; 2) wrapper-based methods, specifically genetic algorithm (GA) optimizing feature subsets through classification accuracy; 3) embedded techniques utilizing deep neural networks (DNN) with autoencoders to extract compact representations; 4) linear projection methods comprising principal component analysis (PCA), linear discriminant analysis (LDA), and partial least squares-discriminant analysis (PLS-DA); and 5) manifold learning through t-SNE for non-linear dimensionality reduction. The optimal feature subsets selected from each modality were subsequently concatenated into a composite feature vector, which was used to train a unified support vector machine classifier. This integrated approach leverages complementary information from multiple sensor sources at the feature level, enabling the classifier to learn from a fused representation that captures the essential characteristics of the samples.

#### 2.5.4 Performance evaluation

Among common classification models including support vector machines (SVM) (Tax and Juszczak, 2002), random forests (Luo et al., 2016), K-nearest neighbors (Kumar et al., 2024), and neural networks (Ju et al., 2022), each presents certain limitations for the current study. Random Forests can be sensitive to parameter configurations, and their predictions may become less reliable when data availability is limited (Zhang et al., 2023), K-Nearest neighbors suffer from performance degradation in high-dimensional spaces (Halder et al., 2024), and neural networks typically require larger datasets for optimal performance (Zhou et al., 2024). In contrast, SVM is particularly well-suited for the medium-scale, high-dimensional data in this study. Its structural risk minimization principle provides inherent protection against overfitting, while the kernel-based mapping enables effective handling of nonlinear class boundaries (Jiang et al., 2020). Furthermore, SVM offers a favorable balance between classification performance and model interpretability. These properties align well with the requirements of our feature-level fusion approach, making SVM the preferred classifier for discriminating samples A, B, and C. Specifically, a multi-class support vector machine (SVM) framework was implemented using error-correcting output codes. To resolve the inherent binary constraint of SVMs, a one-versus-one decomposition strategy was adopted, translating the three-class classification task into multiple binary decision problems. The classifier employed a radial basis function kernel to handle nonlinear pattern separation, with automated kernel scaling. Model generalization was controlled through a box constraint parameter set to 10. This computational framework processes tobacco sample data, ultimately generating classification results for samples A, B, and C. The model evaluation was performed using leave-one-out cross-validation. In this procedure, each sample is sequentially designated as the test set, while the remaining samples constitute the training set, ensuring comprehensive validation across the entire dataset. This approach maximizes the utility of limited sample data and provides a rigorous assessment of model generalization capability.

By using the GC-SAW, E-nose, FTIR data, we establish confusion matrix that is a fundamental tool in the evaluation of classification models, particularly in the context of supervised learning. It provides a detailed breakdown of the model’s performance by comparing the predicted class labels against the truth labels. This matrix is structured as a contingency table, with rows typically representing the true classes and columns corresponding to the predicted classes. In multi-class classification problems, it extends to an N×N matrix, where N denotes the number of classes. This configuration enables a comprehensive analysis of misclassification patterns across all classes, thereby facilitating model refinement and class-specific performance evaluation.

Statistical analysis and graph plotting were performed using Origin 2025 (OriginLab Corp. Massachusetts, USA) and MATLAB R2022b (Mathworks, Natick, USA), respectively. Specifically, data preprocessing, feature extraction, and related model construction were implemented in the MATLAB environment, whereas Origin software was utilized for statistical testing and figure generation.

## 3 Results and discussion

### 3.1 Classification performance of single sensor data

The classification performance of each individual sensor modality was first evaluated to establish a baseline. As summarized in **Supplementary Figure 1**, the models trained on data from a single sensor type exhibited varying levels of accuracy. The E-nose data achieved a classification accuracy of 74.44%, while the model utilizing GC-SAW data yielded a higher accuracy of 83.33%. Notably, the model based solely on FTIR spectra demonstrated perfect separation, attaining a classification accuracy of 100% among the three tobacco shreds samples under the controlled experimental conditions.

### 3.2 Comparison of different data fusion strategies

#### 3.2.1 Results based on data-level fusion

**Figure 3:**
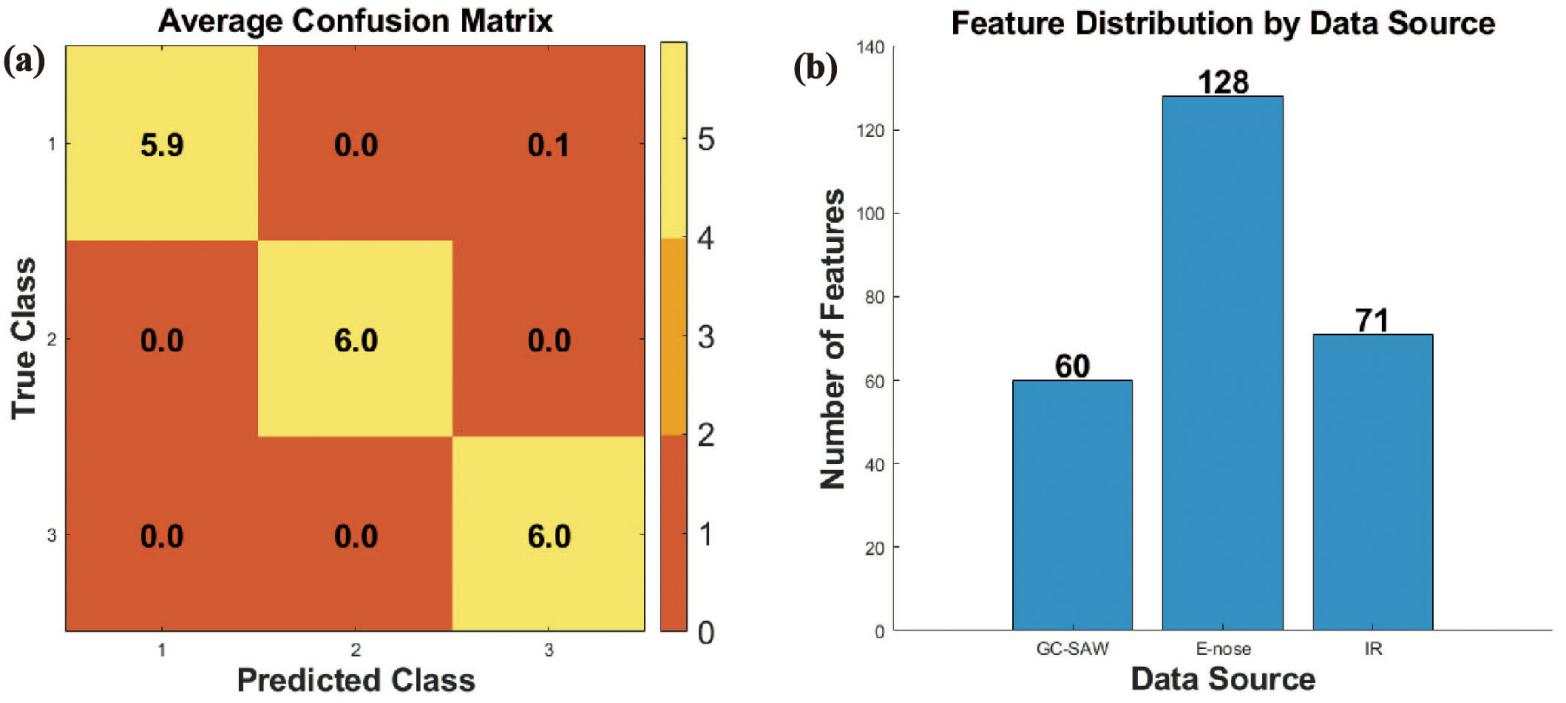
**(a)** Confusion matrix of the data fusion based on the data of FITR, E-nose, and GC-SAW. **(b)** The feature distribution across different data sources.

The data-level fusion approach that involved the direct concatenation of raw feature vectors from the GC-SAW, E-nose, and FTIR sensors, resulted in a high-dimensional dataset comprising 259 features. Classification of this fused dataset using a support vector machine (SVM) yielded a markedly low accuracy of 99.22% ± 2.75% (**Figure 3a**). This performance is substantially inferior to the baseline accuracies achieved by individual sensor, as detailed in Section 3.1.

This significant performance degradation is primarily attributed to the curse of dimensionality (Jiang et al., 2023). The number of features (P = 259) vastly exceeds the number of samples (N = 90) (**Figure 3b**). This scenario critically undermines the effectiveness of the SVM classifier. The high-dimensional space leads to two main issues: firstly, the model is prone to overfitting, capturing noise instead of the underlying generalizable patterns, which severely compromises its generalization capability. Secondly, the process inevitably amalgamates an amount of redundant information and inherent sensor noise from the three instruments, thereby diluting the critical discriminatory information and confounding the classifier. Consequently, data-level fusion theoretically preserves the complete raw information, its practical application in this study is invalidated by the aforementioned challenges, establishing it as an unsuitable strategy for the present dataset.

#### 3.2.2 Results based on decision-level fusion

**Figure 4:**
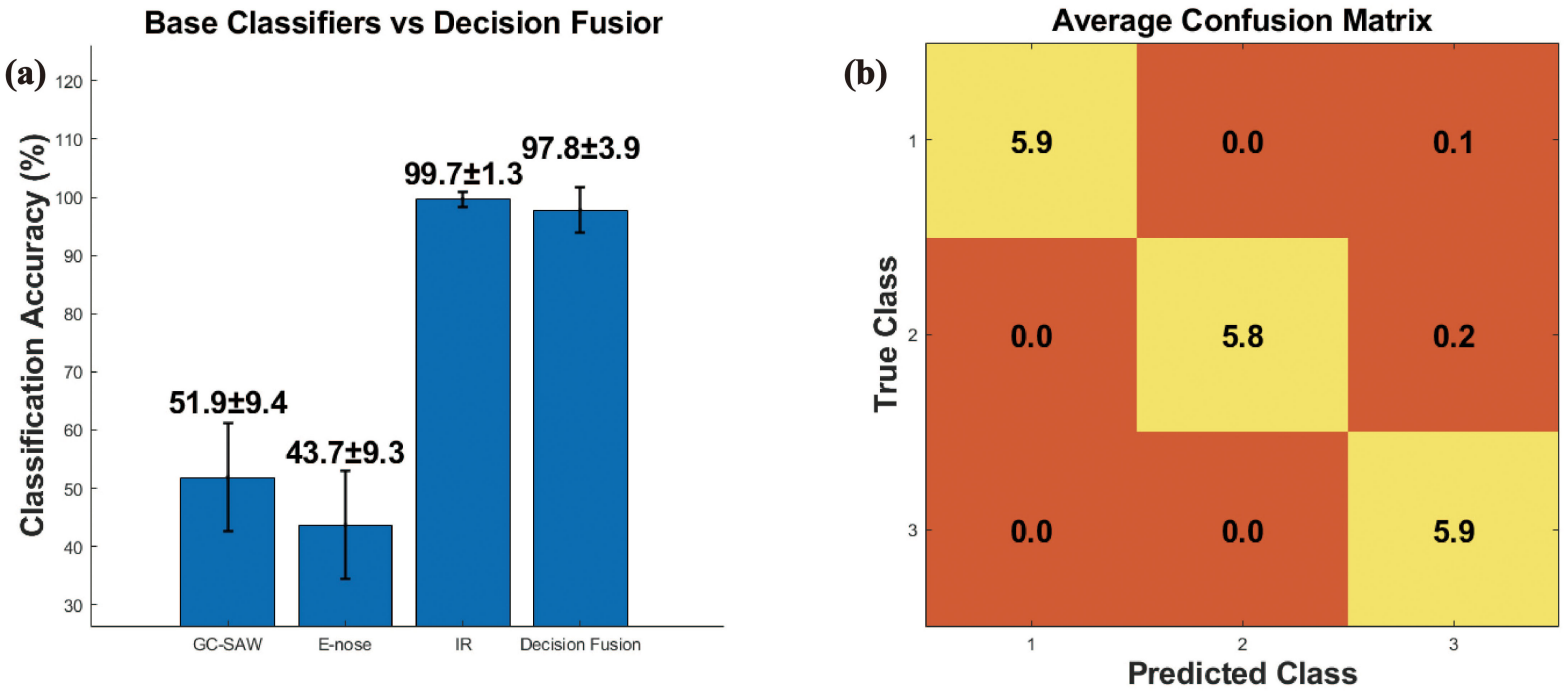
**(a)** The base classifiers and decision fusion. **(b)** Confusion matrix of decision fusion.

The performance of decision-level fusion was evaluated. The base classifiers, when operating on individual sensor data, yielded accuracies of 51.89% ± 9.36% (GC-SAW), 43.67% ± 9.32% (E-nose), and 99.67% ± 1.33% (FTIR) respectively (**Figure 4a**). Notably, the fusion of these decisions via a voting scheme achieved an accuracy of 97.78% ± 3.89%. To address the substantially lower accuracy of GC-SAW and E-nose classifiers compared to FTIR (99.67% ± 1.33%), feature selection should be implemented to improve their discriminatory capability. The result demonstrates that decision-level fusion can synthesize the outputs of multiple classifiers to form relatively accurate collective decision, even when individual ones are potentially weak. The corresponding confusion matrix reveals that misclassifications occurred between class A and C, with 1 samples of A being incorrectly assigned to C, and 1 sample of B being assigned to C (**Figure 4b**). Despite its effectiveness, this approach has an inherent limitation. By operating solely on the final decisions, it discards the rich, complementary information embedded within the original feature sets from each sensor (Sinha et al., 2008). The fusion process does not leverage the potential inter-correlations between features from different modalities, which might be crucial for resolving the observed ambiguities between classes A and C.

#### 3.2.3 Results based on feature-level fusion

**Figure 5:**
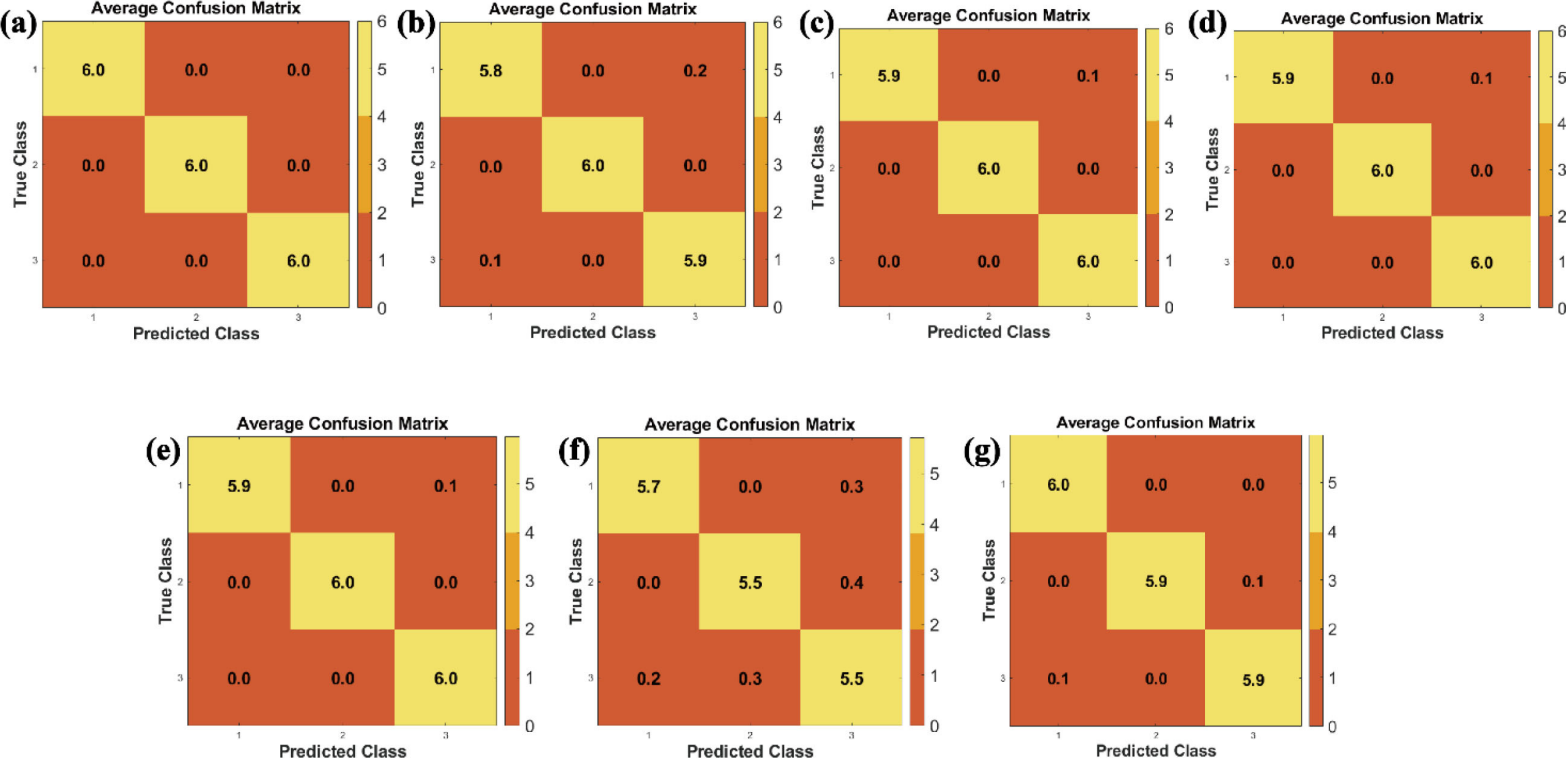
Comparative analysis of the optimal prediction models. Confusion matrices for **(a)** GA, **(b)** LDA, **(c)** PLS-DA, **(d)** PCA, **(e)** RF, **(f)** t-SNE, and **(g)** DNN.

**Figure 6:**
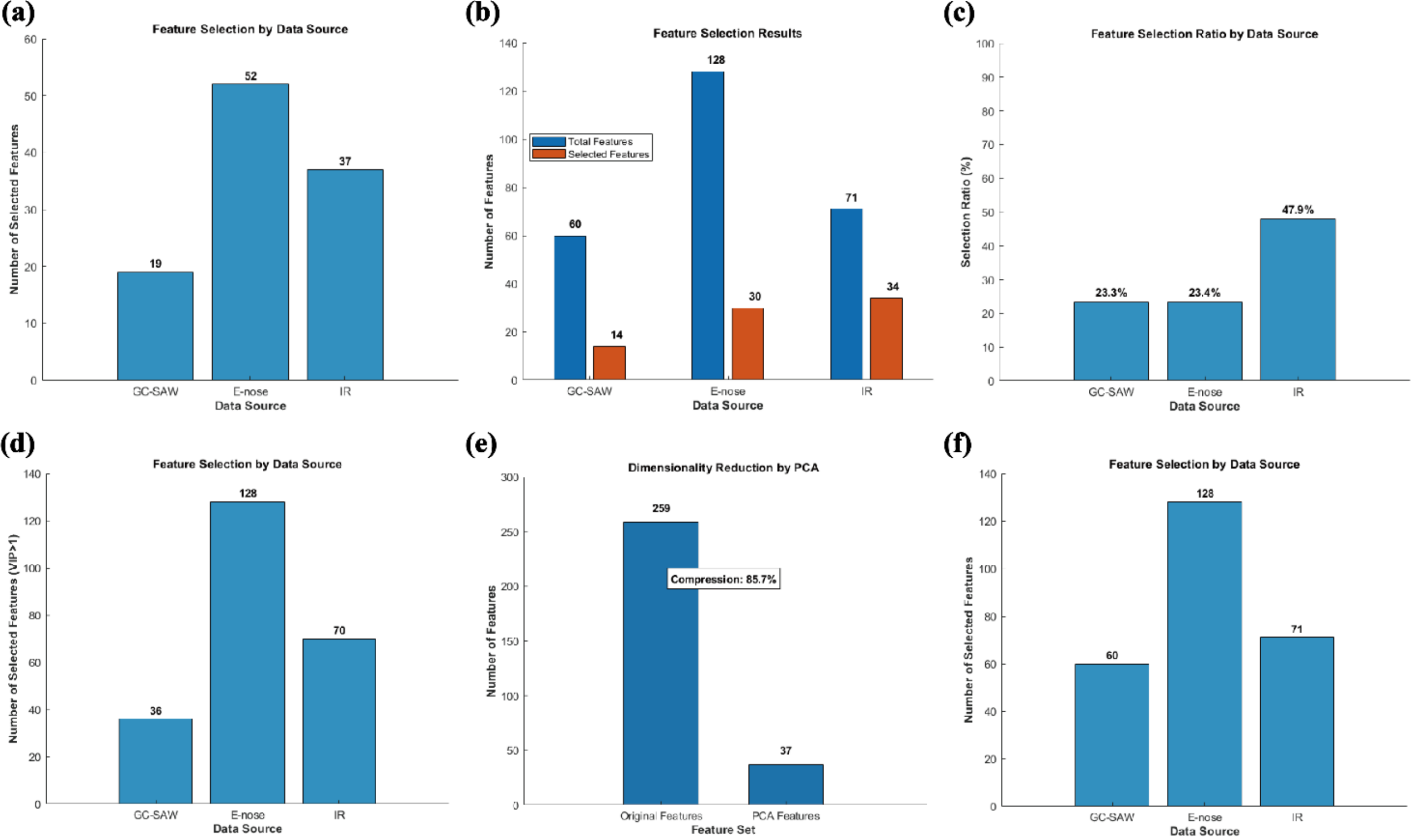
**(a)** Feature selection results using GA. The feature results of LDA **(b)** and **(c)**. The feature selection of PLS-DA **(d)**. The dimension reduction by PCA **(e)**. The feature selection of random forest **(f)**.

Feature-level fusion aims to extract the most discriminative features from the raw data of each sensor separately, followed by concatenating them into a new and information-condensed dataset. Compared with data-level and decision-level fusion, this intermediate approach not only enables the integration of complementary multi-sensor information but also mitigates inherent noise in raw data and ambiguity in preliminary decisions. However, a primary challenge of feature-level fusion is high dimensionality and consequent feature redundancy. Specifically, simply concatenating all features of this study (GC-SAW: 60, E-nose: 128, FTIR: 71) can result in a 259-dimensional feature vector. Given the limited set of only 90 samples, this high dimensionality leads to the curse of dimensionality, consequently causing model overfitting and impaired generalization ability. Hence, an effective dimensionality reduction or feature selection step is crucial to ensure the success of feature-level fusion. To address this issue, we introduce and systematically compare seven dimensionality reduction methods (GA, LDA, PLS-DA, PCA, RF, t-SNE, DNN), aiming to construct a fused feature set and simultaneously addressing its high-dimensional nature. As shown in **Figure 5a**, the GA-based feature selection demonstrated exceptional performance, achieving 99.89% ± 0.79% accuracy under the repeated 5-fold cross-validation. The algorithm selected a compact and informative feature subset (108 features, 41.7% of the original), effectively eliminating redundancy (**Figure 6a**). Notably, the selection was balanced across all sensor modalities (GC-SAW: 31.7%, E-nose: 40.6%, FTIR: 52.1%), indicating that GA successfully identified complementary features from each data source to construct an optimal fused feature set for classification. As shown in **Figure 5b**, LDA feature selection obtained a high accuracy of 98.44% ± 2.98%. It aggressively reduced the feature space to 78 dimensions (30.1% of the raw vector) (**Figures 6b, c**). The feature selection result showed a clear bias, with FTIR features being disproportionately represented (47.9%) in the final subset compared to GC-SAW (23.3%) and E-nose (23.4%) features. These results indicate that the discriminative subspace identified by LDA was primarily defined by the dominant contribution of the FTIR data, which consequently limited the contribution of features from other sensor modalities. The loss of this synergistic information may account for the residual misclassifications (He et al., 2009). As shown in **Figure 5c**, PLS-DA achieved a near-perfect accuracy of 99.67% ± 2.36%. In contrary to other methods, it retained most of the original features (234, 90.3%), with a 100% retention rate for E-nose features (**Figure 6d**). These results indicate that PLS-DA operates more like a feature weighting and projection technique rather than a role of feature selection. Its high performance confirms that the maximum variance in the data is strongly correlated with the class labels, though the model complexity remains relatively high due to the minimal feature elimination. As shown in **Figure 5d**, PCA produced an optimal model with 37 principal components, explaining 95.17% of the total variance, and achieved 99.44% ± 2.57% classification accuracy (**Figure 6e**). This result demonstrates that the majority of discriminatory information within the high-dimensional, heterogeneous feature space can be captured in a much lower-dimensional linear subspace (Cai et al., 2022). PCA effectively denoised the data and created an orthogonal feature set that maximized class separability without requiring explicit feature selection. As shown in **Figure 5e**, the embedded feature importance metric from the random forest classifier was used for feature selection. The model achieved optimal accuracy (99.22% ± 2.75%) using all 259 features and proved resistant to feature reduction based on importance scores (**Figure 6f**). This result indicates that the ensemble method inherently manages high-dimensional spaces by leveraging a broad set of features, where numerous weak contributors collectively support predictions. As shown in **Figure 5f**, t-SNE, primarily a visualization tool, was repurposed for dimensionality reduction, resulting in the lowest accuracy (92.78% ± 6.17%) among the tested methods. Its poor performance is attributed to its non-parametric and stochastic nature, which does not produce a reusable transformation model. The low-dimensional embedding generated is optimized for preserving local neighborhoods rather than global class separation, making it suboptimal for subsequent classification tasks on independent data. As shown in **Figure 5g**, The DNN autoencoder-based approach yielded the accuracy of 98.67% ± 3.65%. This is likely a consequence of the limited sample size (N = 90), which is insufficient for training a complex model with a large number of parameters without severe overfitting (Zhang et al., 2021). Despite regularization attempts (Dropout, L2), the network failed to learn a generalizable representation, highlighting the inadequacy of deep learning techniques for small-scale datasets of this study.

As shown in **Supplementary Table 1**, our comparative analysis reveals that GA represents the optimal approach for feature-level fusion within our framework. Among all methods evaluated using repeated 5-fold cross-validation, GA achieved the highest mean accuracy (99.89%) with the lowest standard deviation (0.79%), indicating superior and more stable performance. GA demonstrated particular merit by performing intelligent feature selection that distilled the most discriminative features from all three sensor modalities in a balanced manner, resulting in a compact, interpretable, and robust feature subset. While PCA also achieved high accuracy (99.44%), it produces transformed features that lack direct physical interpretability. Therefore, GA is identified as the most suitable dimensionality reduction method for enhancing sensing accuracy through feature-level fusion, as it best leverages the complementary information inherent in the multi-sensor data while maintaining model interpretability and stability.

### 3.3 Optimal fusion model

The primary objective of this study is not merely to pursue the highest possible accuracy rate, but rather to construct a more reliable, robust, and interpretable classification system. When conducting single-source data analysis, although FTIR achieved 100% classification accuracy under controlled laboratory conditions, this idea performance covers inherent vulnerabilities such as insufficient generalization capability, sensitivity to measurement conditions, and risks associated with single-point perception. Therefore, we introduce a multi-sensor fusion framework to endow the model with robust and fault-tolerant capabilities. Compared to data fusion and decision fusion algorithms, the feature fusion model based on GA demonstrates better performance than other single techniques.

**Figure 7:**
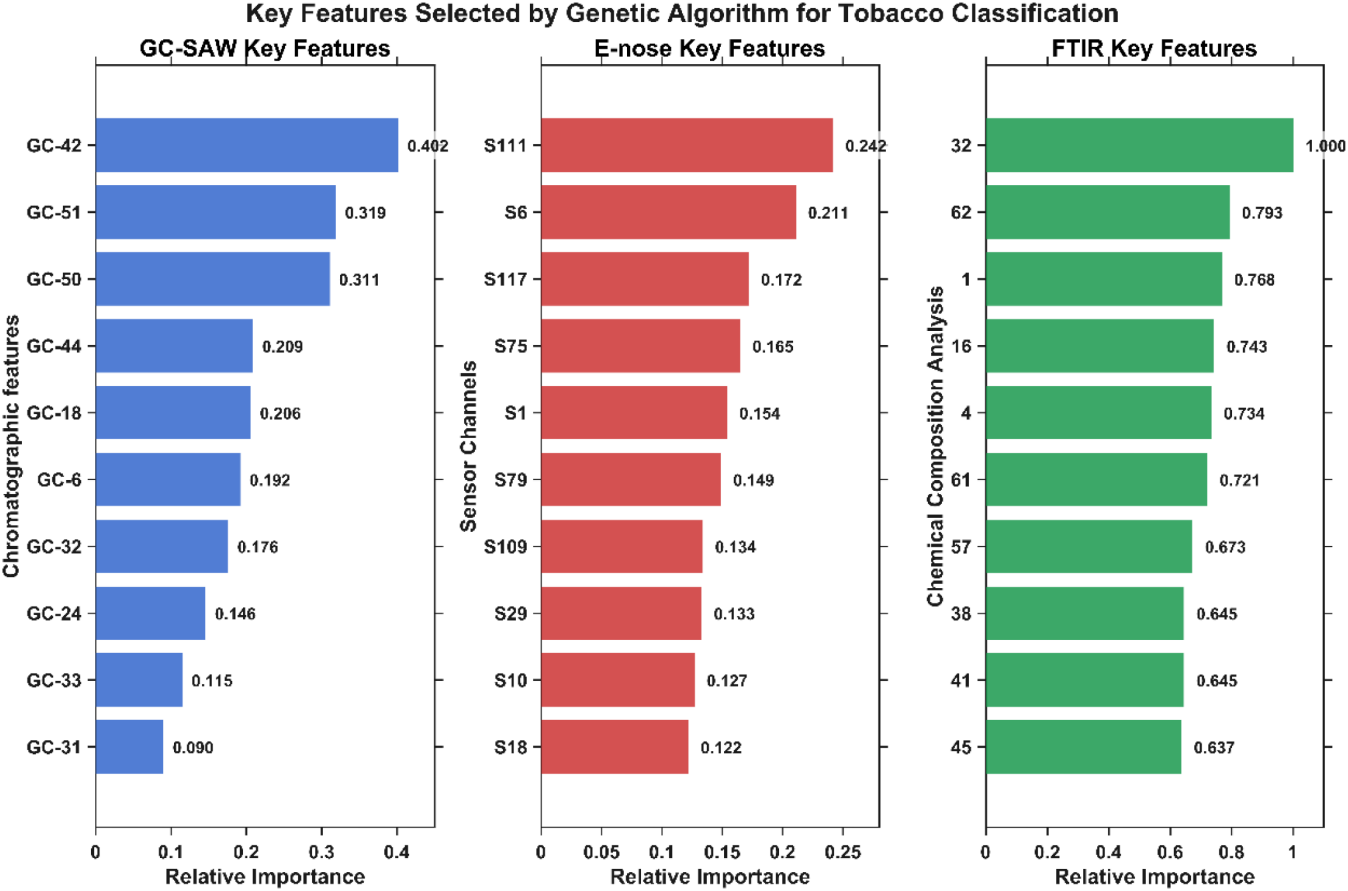
Top 10 features selected by GA across GC-SAW, E-nose, and FTIR platforms, ranked by relative importance.

To elucidate the rationale behind the high performance of the GA-optimized model and to address its interpretability, we analyzed the top-ranked features selected from each sensor modality. **Supplementary Table 2** lists the top 10 features from GC-SAW, E-nose, and FTIR based on their relative importance scores from the GA, along with their potential chemical assignments. The distribution and origin of these key features are further visualized in **Figure 7**.

The analysis reveals that the GA successfully identified a compact yet chemically meaningful set of discriminative features from all three sensors. For GC-SAW, the model heavily relied on specific chromatographic peaks. While several key discriminants (e.g., Peak42, Peak51) correspond to unidentified compounds, the selection of known compounds such as Terpinene (a monoterpene contributing to herbal notes), Butyric acid (a fatty acid influencing aroma), and Butanol (an alcohol) underscores the importance of specific volatile organic compounds related to tobacco flavor and aroma profiles in brand differentiation.

For the E-nose, the GA prioritized a subset of sensors with distinct sensitivities. The top sensors (e.g., Sensor111, Sensor6) are metal-oxide semiconductors with broad cross-sensitivity to complex VOC mixtures. The high importance of Sensor6 and Sensor10 suggests that redox reactions and the presence of specific gases may be indicative of differences in tobacco processing or composition.

Most notably, the FTIR data, processed to quantify specific chemical constituents, revealed that the GA’s selection was related with core tobacco chemistry. The most critical feature was identified as aspartic acid content. Furthermore, the model heavily weighted key metabolites and quality markers, including alkaloid content (a direct measure of nicotine and related compounds), chlorogenic acid (a major polyphenol), and nitrogen content (a fundamental indicator of growth and metabolic activity). The high importance of several fructose-amino acid conjugates (e.g., with aspartic acid, asparagine, and valine) highlights the significance of Maillard reaction precursors and sugar-alkaloid interplay, which are crucial for flavor development and brand-specific characteristics. The selection of amino acids like glycine, cystine, and tyrosine further points to the role of protein metabolism and nitrogen cycle products in discrimination.

The distribution of all selected features (GC-SAW: 19/60, E-nose: 52/128, FTIR: 37/71) demonstrates that the information from the three sensing technologies is highly complementary. The GA did not simply rely on the dominant FTIR signal but intelligently distilled a balanced feature subset that leverages the specificity of GC-SAW for critical volatiles, the pattern-based response of the E-nose to complex odor profiles, and the fundamental quantitative chemical composition provided by FTIR. This synergistic combination collectively constructs a more comprehensive and robust digital fingerprint of the samples.

Ultimately, this GA-optimized model not only achieves high performance but also significantly enhances the interpretability and robustness of the perception system. This transformation stems from its inherent redundancy design and the traceability of key features to concrete chemical properties, moving beyond a “black box” model towards a more transparent and trustworthy analytical tool.

## 4 Conclusions

This study successfully established a robust framework for accurately classifying shredded tobacco by fusing data from GC-SAW, E-nose, and FTIR sensing modalities. The systematic comparison of data-level, feature-level, and decision-level fusion strategies demonstrated the superior potential of the feature-level approach. However, its effectiveness is contingent upon resolving the high-dimensionality challenge inherent in multi-sensor data.

Our key finding is that feature-level fusion, coupled with evolutionary algorithm-based feature selection, constitutes the optimal strategy. The genetic algorithm not only achieved high classification accuracy but also intelligently identified a compact and balanced subset of discriminative features from three types of sensors. This result underscores the value of multi-sensor data fusion, as GA effectively mined the complementary information within the heterogeneous data sources.

Although principal component analysis also achieved 99.44% accuracy, the genetic algorithm-based method demonstrated advantage in providing physically interpretable models. In contrast, methods like PLS-DA and RF achieved high accuracy but failed to substantially reduce feature dimensionality, whereas LDA exhibited a bias towards a single sensor. Techniques such as t-SNE and DNN were found unsuitable for this small-sample-size scenario.

In summary, this work validates that evolutionary algorithm-driven feature selection is a powerful tool for unlocking the full potential of multi-sensor fusion, enabling both high precision and enhanced model interpretability for complex sample classification.

## Data Availability

The raw data supporting the conclusions of this article will be made available by the authors, without undue reservation.
