# Supplementary material for: Evolutionary algorithm-optimized feature fusion for accurate classification of shredded tobacco using multi-sensor data
Source: Front Plant Sci. 2026 Jan 12;16:1728353. doi: 10.3389/fpls.2025.1728353 (PMC12832984; doi:10.3389/fpls.2025.1728353)
Supplement: Supplementary file 1 [file DataSheet1.docx]

Supplementary Material

# Supplementary Figures and Tables

## Supplementary Figures

**(b)**

**(c)**

**(a)**

**
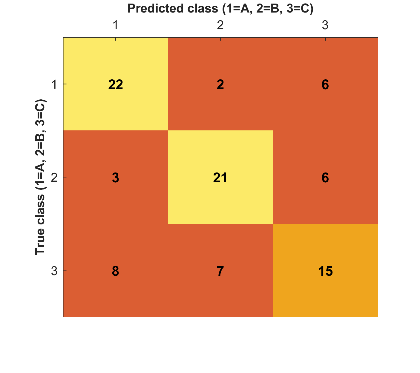
** **
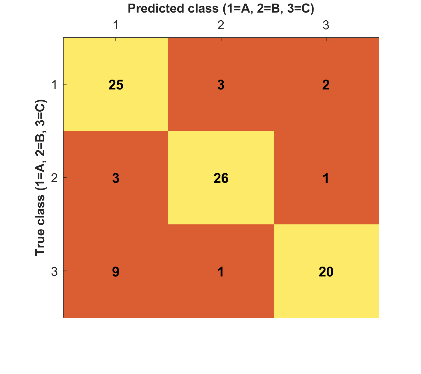
**
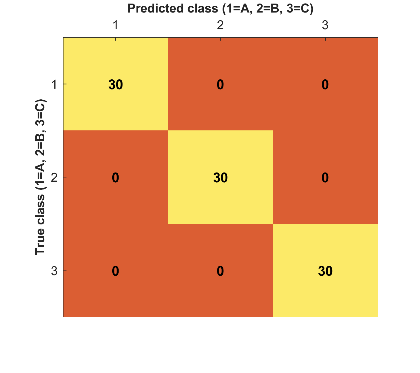
**Figure 1.** **(a)** the confusion matrix of SVM classification toward individual GC-SAW data, **(b)** E-nose data, **(c)** FTIR data, respectively.


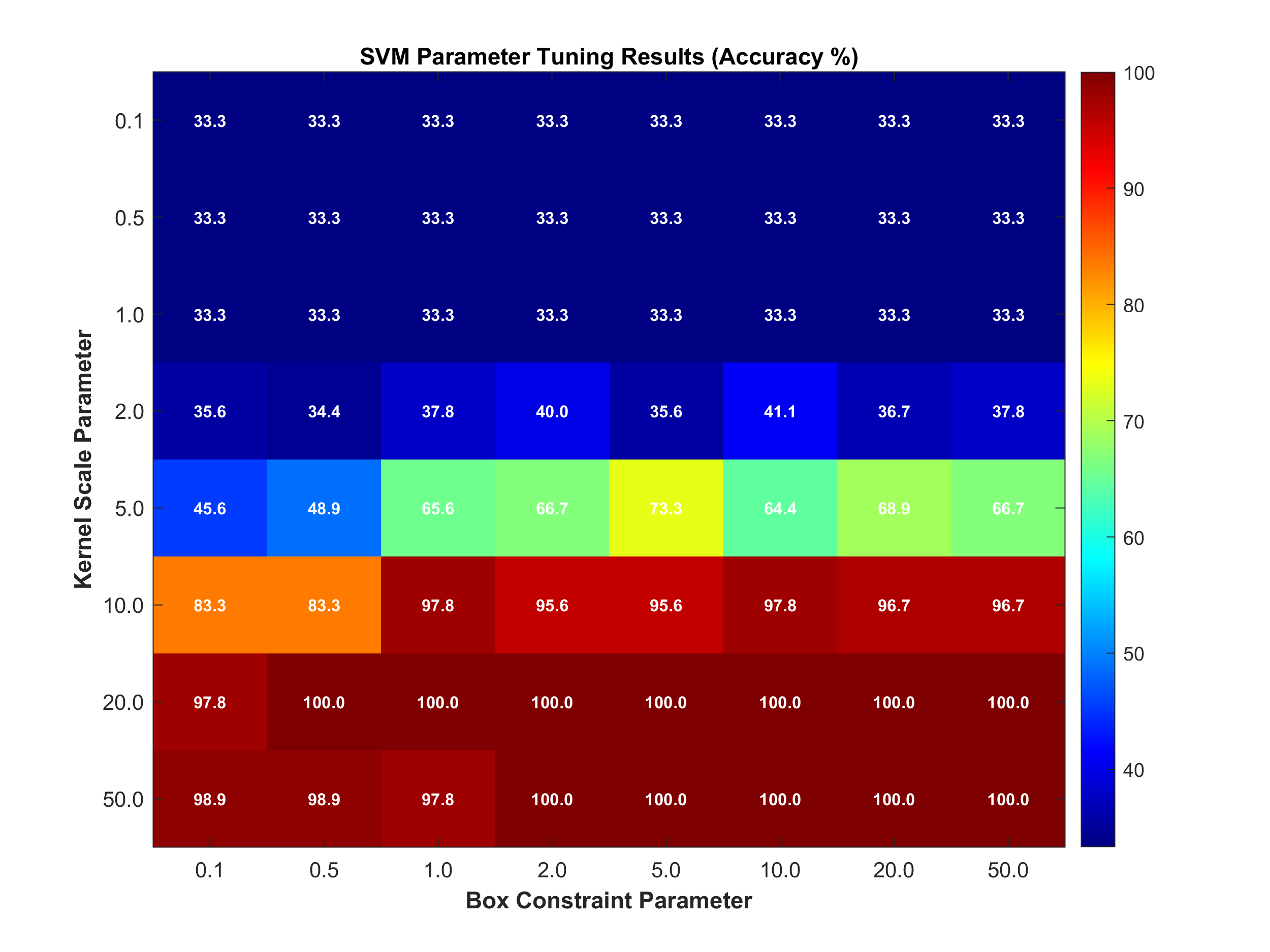


**Figure 2.** The SVM parameters tuning results. The kernel scale is 20 and the box constraint is 0.5.


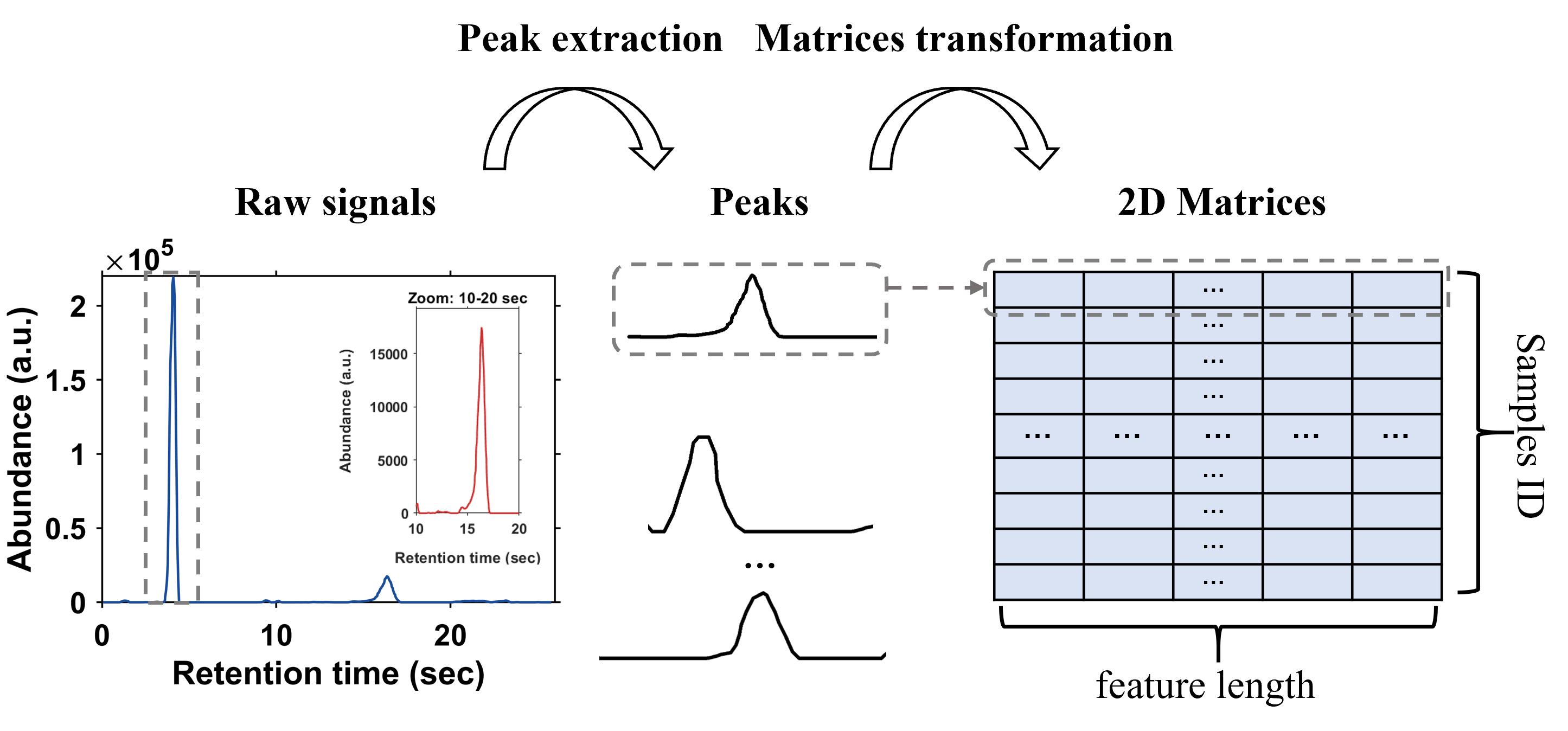


**Figure 3.** The data preprocessing workflow of GC-SAW.

## Supplementary Table

**Table 1.** The parameters of feature fusion

| **Fusion** | **Model** | **Data** | **Parameters** | **Results (100%)** |
| --- | --- | --- | --- | --- |
| **Feature fusion** | GA+SVM | GC-SAW, E-nose, FTIR | Population size=50, Number of generations = 100, Crossover probability = 0.8, Mutationprob = 0.01. | **100** |
|  | LDA+SVM | GC-SAW, E-nose, FTIR | Components=2, Regularization=1e-6, Threshold = 0.3. | 97.78 |
|  | PLS-DA+SVM | GC-SAW, E-nose, FTIR | Components=10, VIP=1. | 98.89 |
|  | PCA+SVM | GC-SAW, E-nose, FTIR | Cumulative variance explained threshold=95. | 100 |
|  | RF+SVM | GC-SAW, E-nose, FTIR | Trees = 100, Minimum number of samples per leaf node=5. | 98.89 |
|  | t-SNE+SVM | GC-SAW, E-nose, FTIR | Target dimensionality after reduction=2, Perplexity parameter=3, Number of optimization iterations=1000. | 88.89 |
|  | DNN | GC-SAW, E-nose, FTIR | Input layer dimensionality=259, Number of neurons in hidden layer=24, Output neurons=3, learning rate =1e-4, Epochs=1000, Dropout rate for regularization = 0.4, L2 regularization coefficient=0.01. | 77.78 |
| Data fusion | SVM | GC-SAW, E-nose, FTIR | Regularization parameter C = 15. | 26.67 |
| Decision fusion | SVM | GC-SAW, E-nose, FTIR | Regularization parameter C = 10, 15, 20, respectively. | 95.6 |

**Table 2.** Top 10 features selected by GA

| Rank | Sensor Source | Feature Identifier | Relative Importance |
| --- | --- | --- | --- |
| 1 | GC-SAW | GC_Peak42 | 0.402 |
| 2 | GC-SAW | GC_Peak51 | 0.319 |
| 3 | GC-SAW | GC_Peak50 | 0.311 |
| 4 | GC-SAW | GC_Peak44 | 0.209 |
| 5 | GC-SAW | GC_Peak18 | 0.206 |
| 6 | GC-SAW | GC_Peak6 | 0.192 |
| 7 | GC-SAW | GC_Peak32 | 0.176 |
| 8 | GC-SAW | GC_Peak24 | 0.146 |
| 9 | GC-SAW | GC_Peak33 | 0.115 |
| 10 | GC-SAW | GC_Peak31 | 0.09 |
| 1 | E-nose | Sensor111 | 0.242 |
| 2 | E-nose | Sensor6 | 0.211 |
| 3 | E-nose | Sensor117 | 0.172 |
| 4 | E-nose | Sensor75 | 0.165 |
| 5 | E-nose | Sensor1 | 0.154 |
| 6 | E-nose | Sensor79 | 0.149 |
| 7 | E-nose | Sensor109 | 0.134 |
| 8 | E-nose | Sensor29 | 0.133 |
| 9 | E-nose | Sensor10 | 0.127 |
| 10 | E-nose | Sensor18 | 0.122 |
| 1 | FTIR | Feature32 (Aspartic Acid) | 1 |
| 2 | FTIR | Feature62 (Fructose-Aspartic Acid Conjugate) | 0.793 |
| 3 | FTIR | Feature1 (Alkaloid Content) | 0.768 |
| 4 | FTIR | Feature16 (Chlorogenic Acid) | 0.743 |
| 5 | FTIR | Feature4 (Nitrogen Content) | 0.734 |
| 6 | FTIR | Feature61 (Fructose-Asparagine Conjugate) | 0.721 |
| 7 | FTIR | Feature57 (Fructose-Valine Conjugate) | 0.673 |
| 8 | FTIR | Feature38 (Glycine) | 0.645 |
| 9 | FTIR | Feature41 (Cystine) | 0.645 |
| 10 | FTIR | Feature45 (Tyrosine) | 0.637 |
